# The efficacy of oral brush biopsy with computer-assisted analysis in identifying precancerous and cancerous lesions

**DOI:** 10.1186/1758-3284-3-39

**Published:** 2011-08-24

**Authors:** Ravi Mehrotra, Sanjay Mishra, Mamta Singh, Mangal Singh

**Affiliations:** 1 Department of Pathology, Moti Lal Nehru Medical College, Lowther Road Allahabad, 211001 India; 2 Department of Otorhinolaryngology, Moti Lal Nehru Medical College, Lowther Road Allahabad, 211001 India

**Keywords:** Oral, Brush, Biopsy, Precancer, Cancer, Diagnosis, Efficacy

## Abstract

**Background:**

Cancer of the oral cavity is the sixth most common malignancy reported worldwide and one with the highest mortality rate among all malignancies. There is a paucity of reliable diagnostic methods to detect early malignancies. This study was performed to evaluate the sensitivity and specificity of brush biopsy in identifying oral premalignant and malignant lesions.

**Methods:**

Oral brush and scalpel biopsies were performed on 85 consecutive patients presenting with an oral lesion deemed to be minimally suspicious by clinical examination and the results were compared.

**Results:**

Of 79 patients with adequate brush biopsy samples with matching scalpel biopsies, 27 revealed histopathologic evidence of dysplasia or carcinoma, 26 of which were independently identified with the oral brush biopsy (sensitivity: 96.3% - 95% CI, 87%-100%). 52 oral lesions did not reveal any histopathologic evidence of dysplasia or carcinoma and of these, brush biopsy reported 47 as "negative" and 5 as "atypical"(specificity of "positive" brush biopsy result is 100%- 95% CI, 93%-100%; specificity for "atypical" brush biopsy result is 90.4%- 95% CI, 82%-97%. The positive predictive value of an abnormal oral brush biopsy was 84% and the negative predictive value was 98%.

**Conclusion:**

Our study demonstrated that the oral brush biopsy is an accurate test in identifying oral premalignant and malignant lesions, even if minimally suspicious.

## Background

Cancer of the oral cavity is the sixth most common malignancy reported worldwide and one with the highest mortality rate among all malignancies. In 2011, an estimated 34,300 patients developed oral cavity and oropharyngeal cancer in the United States, and approximately 6900 died from the disease [1]. In India, oral cancer represents a major health problem accounting for up to 40% of all cancers, and is the most prevalent cancer in males and the third most prevalent cancer in females [2,3]. Joseph et al. reported that early diagnosis of oral cancer greatly increases the probability of cure with minimum impairment and deformity [4]. Primary prevention, which involves adopting a healthy lifestyle and reducing the exposure to tobacco, alcohol and betel quid has been shown to be effective in reducing the incidence of oral cancer. Secondary prevention involves detecting and controlling early stage cancer and the precursor stage- dysplasia, when such lesions are easiest to cure [4]. The implementation of both primary and secondary prevention programs is essential to reduce the high oral cancer mortality rate.

One major problem inherent in current oral cancer screening is that visual inspection often cannot differentiate between lesions harboring dysplasia and/or early cancer from those that do not. This is especially true for innocuous looking lesions which are subjected to "watchful waiting" and close follow-up despite the fact that some precancerous and cancerous cells within them remain undetected and are allowed to progress to a more advanced stage [5]. The practice of not properly evaluating all suspicious lesions, that is, lesions without a specific etiology such as trauma or infection, invariably results in delay of the correct diagnosis, limiting treatment options.

Another obstacle in the early detection of oral cancer and precancer is the fact that a large proportion of the population- up to 15%- have an oral lesion [6,7]. Not only is it impractical to subject all of these lesions to a surgical biopsy but the procedure itself is associated with pain and morbidity. Furthermore, when presented with the need to have an oral biopsy performed, patients are often understandably reluctant to undergo an invasive surgical procedure. This may be compounded by the clinician's hesitation to perform a surgical procedure in an unfamiliar anatomical site. Additionally, in US medical schools and dental schools, training in oral cancer identification is often inadequate [8], and in one study of senior dental students at three Texas dental schools, just over 50% had ever observed an oral biopsy and only about 25% had actually performed one [9]. Not surprisingly, only a quarter of leukoplakias, the most common oral precancer, are ever subjected to biopsy [10]. Given the limited value of an oral examination as a method for detecting precancerous or early cancerous lesions, additional diagnostic tests are desperately needed.

In contrast to sampling cells of the uterine cervix, analysis of surface epithelial cells of the oral cavity and oropharynx by standard exfoliative cytology has proven unreliable [11]. Without loss of minimal invasiveness, it is not possible to access the deeper cell layers of the oral cavity with conventional exfoliative cytology [12].

The oral brush biopsy with computer-assisted analysis is simple to perform, non-invasive, and has the potential to overcome many of the obstacles that have hindered early detection of early stage cancers and dysplasia [13]. In published studies in which oral lesions were subjected to both brush biopsy and scalpel biopsy concomitantly, the brush biopsy was found to have a sensitivity and a specificity of greater than 90% in identifying dysplasia and carcinoma [14,15].

Our aim was to evaluate computer-assisted analysis of oral brush biopsies in a screening program that replicates its use in an office setting of dentists and physicians. We tested oral lesions that did not have an obvious etiology and were minimally suspicious clinically, and compared the results of brush biopsy with scalpel biopsy to determine the brush biopsy's diagnostic accuracy and usefulness.

## Methods

Patients who were at least 18 years of age presenting with unrelated complaints to the outpatient Department of Otorhinolaryngology, Moti Lal Nehru Medical College in Allahabad, were screened by a team of specialists and residents-in-training between July and November 2010. We obtained clearance from the Institutional ethics committee, and written consent was also granted by all patients.

**Figure 1:**
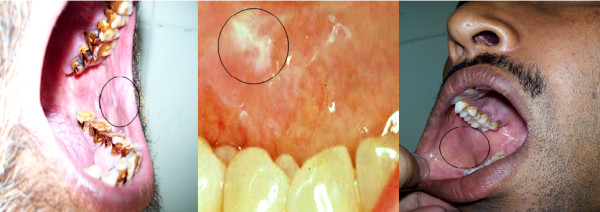
**Clinical examples of minimally suspicious lesions sampled**.

Patients with an oral epithelial abnormality that appeared clinically benign- minimally suspicious- and did not have an obvious etiology such as trauma or infection were prospectively enrolled in the study (Figure 1). The inclusion and exclusion criteria for patients enrolled in the study are summarized in Table 1.

**Table 1:** Inclusion and exclusion criteria for patients enrolled in the study

Inclusion criteria	Exclusion criteria
Patients with an oral epithelial abnormality that appeared clinically benign- minimally suspicious- without any obvious etiology such as trauma or infection.	Patients with medical issues and dental appliances such as orthodontic or other fixed prostheses that could interfere with the examination were excluded.
Multiple oral lesions.	Oral lesions suggestive of dysplasia or cancer were excluded.
	Patients with oral lesions that were either submucosal (i.e. cyst or salivary gland tumor) or covered with a clinically intact normal epithelium (i.e. hemangioma or fibroma).
	Pigmented lesions such as nevi and amalgam tattoos as well as lip lesions, specifically on the vermilion border or cutaneous surfaces,

Demographic information of each patient was obtained including the patients' age, sex, and consumption of tobacco and alcohol. With regard to tobacco consumption, patients were classified non-users if they 1) reported no use and 2) stopped tobacco use 10 or more years prior to the study. We classified tobacco users as 1) persons who stopped using tobacco less than 1 year prior to the study and 2) ongoing users. Patients were considered alcohol users if they consumed more than an average of 1 drink per day for at least a year.

Prior to the examination, patients rinsed their mouth thoroughly with water. The location, size and colour of each lesion was ascertained from a thorough oral examination and documented. Every patient underwent a brush biopsy and then a scalpel biopsy concomitantly at the same visit.

### Oral Brush Biopsy

A specially designed brush was used to obtain a transepithelial specimen from all patients. The material from the brush was spread on clean and dried glass slides, fixed immediately, and sent for further processing to OralCDx Laboratories^® ^(Suffern, New York, USA) where the results were determined in a blinded fashion, independent of the scalpel biopsy results. The brush biopsy results were classified into one of three categories as follows: "negative" - no epithelial abnormality; "atypical" - abnormal epithelial changes; "positive" - definitive evidence of epithelial dysplasia or carcinoma. Using accepted reference standards for cytologic evaluation, atypical and positive results are considered abnormal as both warrant histologic analysis. Patients with incomplete specimens- those that did not demonstrate cells from all layers of the epithelium- were excluded from the study and data analysis. Training of investigators consisted of providing verbal instructions and watching a brief training video for performing an oral brush biopsy.

### Scalpel biopsy

After the brush biopsy was performed, the same investigator performed a scalpel biopsy of the lesion and in the same location tested with the brush biopsy. After routine processing and paraffin embedding, several sections (3-4 μm thickness) were cut and stained with Hematoxylin and Eosin and then examined by a pathologist in a blinded fashion independent of the brush biopsy results.

**Figure 2:**
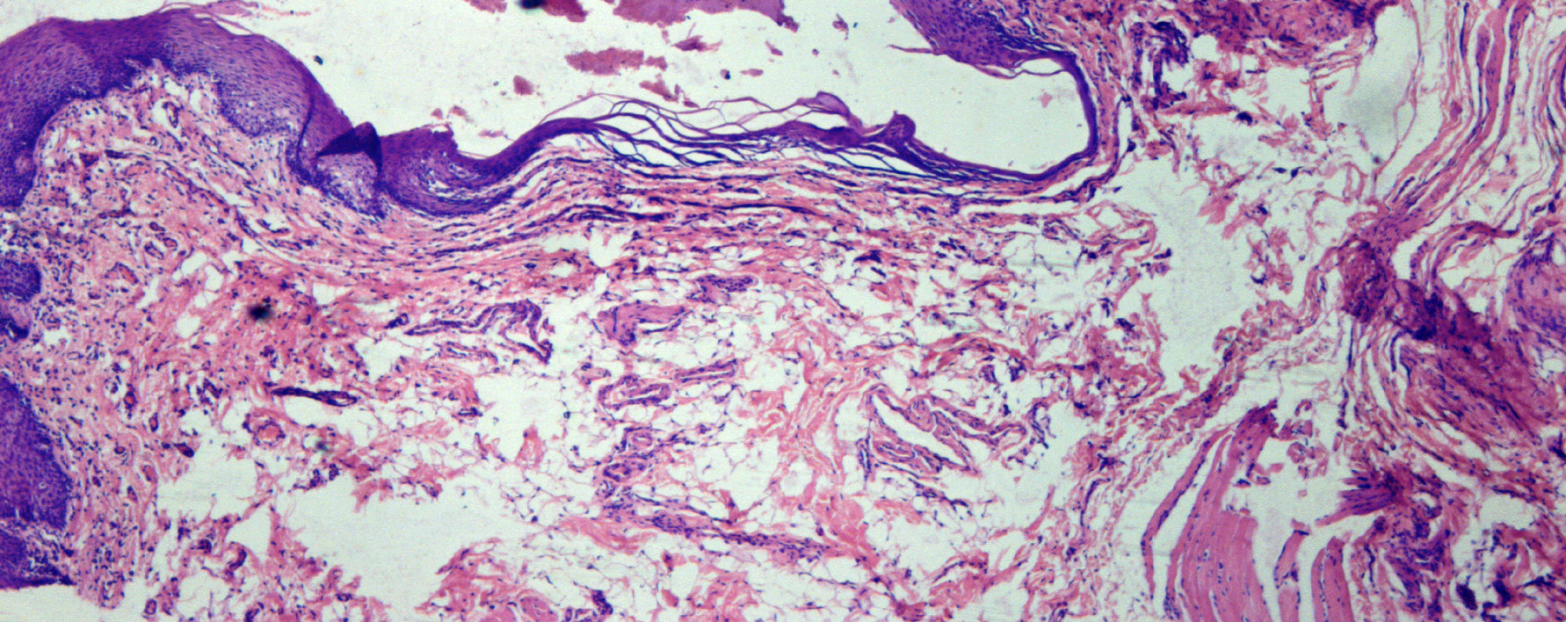
**Histopathologic specimen demonstrating the oral biopsy defect sampling the entire thickness of the epithelium**. (H &E x100).

In patients with discrepant oral brush biopsy and scalpel biopsy results, we confirmed the presence of the brush biopsy tissue defect in the histological specimen of the scalpel biopsy. This ensured that the same part of the lesion was indeed sampled by both the brush and the scalpel instruments. (Figure 2)

Statistical confidence intervals (CI) were calculated based on Student's t-distribution and the exact binomial Clopper-Pearson interval. Data analysis used the Mathematica software package^® ^(Champaign, USA).

## Results

Among 820 subjects consecutively screened, 85 patients (M: F 55:30) were found to have a minimally suspicious-appearing white or red spot that did not have an etiology. The mean age at presentation was 45.5 with an overall age range of 25 to 75 years. More than 50% of the 85 patients consumed either tobacco or alcohol or both. The profile of the study patients are presented in Table 2.

**Table 2:** Profile of study patients (n = 85)

Sex ratio (M/F)	1/8:1 (55/30)
Age range:	25 - 75 (Mean: 45.5)
Tobacco use *	37 (44%)
Alcohol use **	9 (11%) (8-21 drinks/week)
Both tobacco and alcohol	10 (12%)

*Tobacco users: 1) persons who stopped using tobacco less than 1 year prior to the study and 2) ongoing users.

** Alcohol users: patients who consumed more than an average of 1 drink per day for at least a year.

The buccal mucosa was the most common site of involvement accounting for just less than 50% of all cases, followed by the tongue, alveolar mucosa and gingivae.

The lesions demonstrated a wide range of clinical characteristics and are presented in Table 3.

**Table 3:** Clinical characteristics of lesions tested by both the brush biopsy and scalpel biopsy (n = 85)

Predominant Colour	
White:	49
Red	18
Mixed	11
Not specified:	7
	
**Location**	
Buccal mucosa	38
Tongue/Floor of mouth	18
Alveolar and labial mucosa	8
Gingiva	8
Hard palate	7
Site unspecified	6
	
**Size**	
Less than 5 mm	45
5-10 mm	20
10-20 mm	3
> 20 mm	0
Not specified	17

There were 6 patients (7%) with an inadequate brush biopsy sample who were excluded from the study. The results of 79 patients with matching and adequate brush biopsy and scalpel biopsy samples were analyzed.

Of the 79 patients, 27 revealed histopathologic evidence of dysplasia or carcinoma. The brush biopsy independently detected all of these cases with the exception of one patient with dysplasia. The sensitivity rate, defined as a measure of the likelihood that a patient with dysplasia or carcinoma will have an abnormal brush biopsy result was 96.3% (26/27); 95% CI, 87%-100%. An additional 52 oral lesions, which were evaluated histologically, did not reveal any features of dysplasia or carcinoma. Of these, the brush biopsy reported 47 as "negative" with no epithelial abnormalities and 5 as "atypical". The specificity rates, defined as a measure of the likelihood that a patient with a benign lesion will have a "negative" result is 100% (52/52) 95% CI, 93%-100% for "positive" brush biopsy results and 90.4% (47/52) 95% CI, 82%-97% for "atypical" brush biopsy results. The results of all oral brush biopsy and histopathologic diagnoses are summarized in Table 4.

**Table 4:** Results of brush biopsy and scalpel biopsy

N = 79	Scalpel Biopsy Malignant or Dysplastic	Scalpel Biopsy Benign	Total
Brush Biopsy Positive	1	0	1
Brush Biopsy Atypical	25	5	30
Brush Biopsy Negative	1	47	48
Total	27	52	79

**Sensitivity **of brush biopsy: 96.3%; 95% CI, 87%-100%).

**Specificity **of "positive" brush biopsy result: 100%; 95% CI, 93%-100%

**Specificity **for "atypical" brush biopsy result: 90.4%; 95% CI, 82%-97%

**Positive predictive value **of an abnormal brush biopsy: 84%

**Negative predictive value **of an abnormal brush biopsy value: 98%.

**Figure 3:**
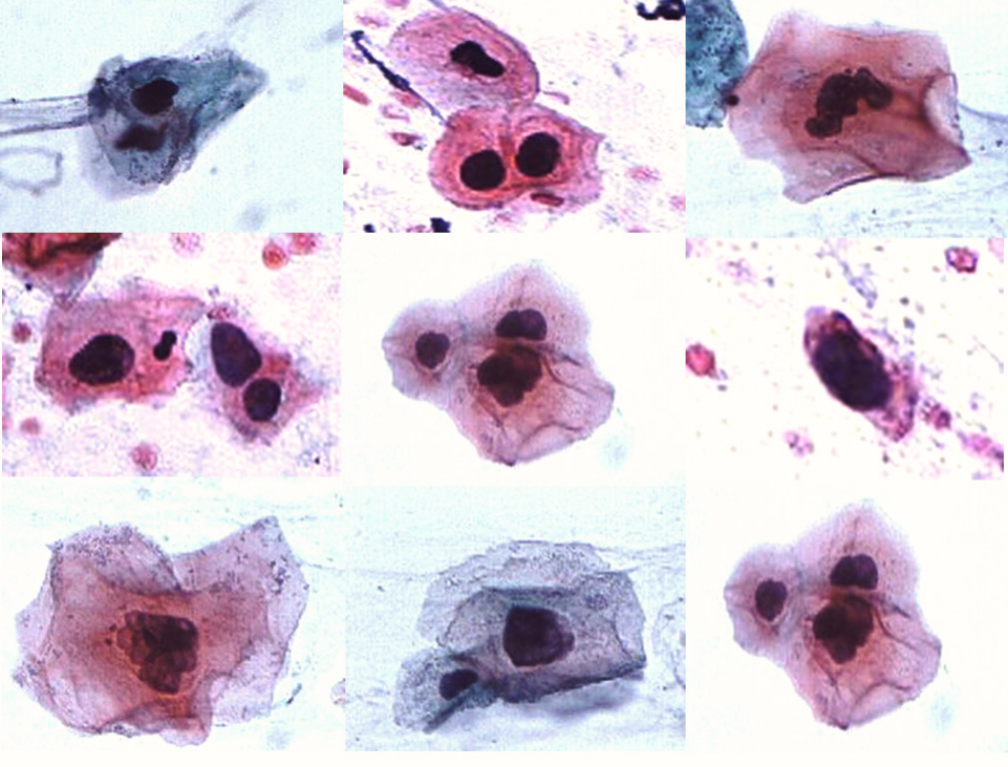
**Panorama of atypical and malignant cells identified from a brush biopsy specimen with the aid of a highly specialized neural network-based image-processing system**. (Pap × 1000).

Of the 79 patients, 31 had abnormal brush biopsy results (Figure 3) and of these, histology from 26 cases was positive for either dysplasia or carcinoma. The positive predictive value of an abnormal brush biopsy, defined as a measure of the likelihood that a patient with an abnormal brush biopsy will have a histologic report of dysplasia or carcinoma on follow-up scalpel biopsy was 83.7% (26/31) and the negative predictive value was 98% (47/48).

## Discussion

In two recent review articles on oral cancer diagnostic aids, Patton et al. [16] and Lingen et al. [17] concluded that based upon published studies, oral brush biopsy with computer-assisted analysis has been demonstrated to be valuable for detecting dysplasia and cancer when evaluating "clinically suspicious" lesions. Both groups suggested that the accuracy of the oral brush biopsy for testing "minimally suspicious" lesions has not yet been established. Our study indisputably demonstrates that the brush biopsy is as sensitive and specific for evaluating "minimally suspicious" lesions as well as "suspicious" lesions and makes it an effective test to evaluate the entire spectrum of lesions detected during an oral cancer screening examination.

The accuracy of the oral brush biopsy has been questioned by some authors who reported lower sensitivity and specificity results compared to those reported in our study [18-20] Purported oral brush biopsy "false-negative" or "false positives" cases in the literature are not a reliable comparison of the efficacy of the brush biopsy vs. the scalpel biopsy since these discrepant anecdotes, which have been quoted repeatedly in the literature [16,17], are of questionable value. In almost all cases, discrepant results were reported from patients who had a scalpel biopsy and brush biopsy performed at widely different times- often months or over a year apart. Within a given oral lesion, dysplasia is multicentric and therefore, unless the 2 biopsies test the same part of the dysplastic lesion by chance, the results will often be discrepant. Furthermore, the biologic nature of a lesion may change over time as benign lesions may become dysplastic and dysplasia may also regress [21]. Finally, the histologic diagnosis of dysplasia is not easily reproduced amongst oral pathologists, with poor intraobserver and interobserver variability in the diagnosis of oral dysplasia [22,23]; therefore, a discrepant result between brush biopsy and scalpel biopsy may also, in fact, represent a false negative or false positive scalpel biopsy result.

The limitations of comparing any two biopsy results performed at different times are highlighted in a study of 200 patients with leukoplakia [24]. In this study, scalpel biopsies were obtained from the same lesion at different times, and the results showed an agreement rate between two scalpel biopsies of only 56%. In another study by Holmstrup et al [25], the degree of dysplasia in biopsies from 101 oral lesions was compared with the results from the entire specimen which was examined in step sections, and the concurrent diagnosis was achieved in only 49% of cases. As others have noted, when comparisons are made between any two biopsy techniques (i.e. brush biopsy vs. scalpel biopsy or scalpel biopsy vs. scalpel biopsy), only studies comparing the results of both biopsies performed at the same time and from the same portion of the suspicious lesion should be considered valid [26,27]. In the 3 studies in which an oral lesion was simultaneously tested with both a brush biopsy and scalpel biopsy, including our study, the oral brush biopsy with computer assistance has been shown to have a sensitivity and specificity well over 90%.

For purposes of determining specificity, it is assumed that when the brush biopsy detects dysplasia or cancer which is not found on the scalpel biopsy, such cases are classified as brush biopsy "false positives." Although histology is the standard for diagnosis, as with all anatomic pathology laboratories, histologic sampling is not without errors, and false positives as well as false negatives do occur [28]. In our study, there were 5 patients whose brush biopsies were abnormal and whose scalpel biopsies were negative. It is possible that several cases of dysplasia or cancer may have been missed with the scalpel biopsy. Under ideal circumstances, these patients would be recalled and a second scalpel biopsy would be performed.

In another study, brush biopsies and scalpel biopsies were performed concomitantly with the authors reporting several brush biopsy false negatives in identifying dysplasia, however, all cancers were identified with the brush biopsy [20]. As the authors in that study point out, confirmation of a brush biopsy false negative result requires a brush biopsy tissue defect to be present in the histological specimen of the scalpel biopsy to ensure that the same part of the lesion was sampled by both biopsies. Yet in that study, not a single example of matched brush and scalpel biopsies was presented for any of the purported discrepant results. Therefore, a likely conclusion is that different portions of the lesion may have been sampled. Furthermore, surprisingly, 43 of the 69 lesions in that study were presumably dysplastic and 15 were frank cancers, despite the fact that all of the lesions in the study were not clinically suspicious. Bukhardt has analysed their results and found some glaring problems supporting the notion that the authors' claim of finding dysplasia on brush biopsy negative specimens likely represents false positive histology results [29].

The high positive predictive value (PPV) of our study is in agreement with 5 other studies published [14,15,19,28,30] and contrasts with the results of a study by Bhoopathi et al. who reported a low PPV [31]. In their study, the authors claim that 3 "positive" brush biopsy specimens were negative on histology even though "positive" brush biopsy cases always display cellular features pathognomonic for dysplasia or carcinoma; therefore, it is doubtful that these cases represent actual false positive brush biopsy results.

The presence and number of basal cells in the brush biopsy specimen, as detected by both the computer and the examining pathologist, is the standard used by the laboratory to determine the adequacy of the sample. In other published clinical trials, incomplete brush biopsy results with an insufficient basal cell count have been reported between 2% and 7%. The incomplete rate in our study was 7% and within the reported range of other studies. Our rate of incompletes could be due to the fact that our study was conducted by residents-in-training. As their experience with the technique improved, the number of incomplete samples decreased.

The brush biopsy kits used in our study include both a proprietary brush and an analysis with neural network computer assistance of the specimens. Researchers, including the author's group, have attempted to use the brush biopsy instrument without computer assistance [32], the same brush in a liquid based preparation [33], a different brush with analysis of specimens using computer assistance [34], and a variety of so called instruments or "brush biopsies" that collect a complete epithelial sample. When subjected to studies, however, all of these proved to have an unacceptably poor sensitivity and specificity. As highlighted in many studies [14,35,36], the combination of a transepithelial brush specimen and neural network analysis of that specimen are necessary to ensure an accurate result.

The limitations of the current study include: (a) not being able to recall and re-biopsy patients with abnormal brush biopsies and negative scalpel biopsies to determine if these represented false negative scalpel biopsy results; and (b) "minimally suspicious" lesions which were included in the study are highly subjective, and what may appear to be suspicious to one observer may not be suspicious to others who examine the same lesion. Although we did not calibrate the examiners as to which lesions were minimally suspicious, the outcome of the study would not likely to have changed; (c) since this study was carried out in a location in India with a high prevalence of oral lesions, the results should be evaluated with care.

## Conclusion

All persistent white and red lesions that do not have an obvious etiology such as trauma or infection require evaluation- and not "watchful waiting". Failure to conform to the standard of care, which requires all unexplained lesions to be evaluated, can have dire consequences for both the patient and the oral care provider [37]. The results of our study, the first in the literature where matched oral brush and scalpel biopsies were performed simultaneously on patients with minimally suspicious oral lesions demonstrate that the computer-assisted analysis of a brush biopsy is a highly sensitive and specific, noninvasive test in the evaluation of all oral lesions without an etiology. The test is especially beneficial when used on lesions that appear clinically benign for identifying early stage cancers and dysplasias - the lesions for which therapy is most effective. As an adjunct to oral cancer examination, its use has the potential to reduce the poor mortality rate associated with oral malignancies.

## Competing interests

The authors declare that they have no competing interests.

## Authors' contributions

RM was responsible for conception and design of the study and finalized the manuscript, SM conducted the procedures and analyzed the results, MS did the evaluation and finalized the manuscript, MS provided the clinical details and biopsy confirmation. All authors read and approved the final manuscript.

## References

[B1] http://www.cancer.org/Cancer/OralCavityandOropharyngealCancer/DetailedGuide/oral-cavity-and-oropharyngeal-cancer-key-statistics Last Accessed Jul 1, 2011

[B2] Sankaranarayanan R Oral cancer in India: an epidemiologic and clinical review Oral Surg Oral Med Oral Pathol 1990 69 3 325 330 10.1016/0030-4220(90)90294-3 2179801

[B3] Mehrotra R Pandya S Chaudhary AK Kumar M Singh M Prevalence of oral pre-malignant and malignant lesions at a tertiary level hospital in Allahabad, India Asian Pac J Cancer Prev 2008 9 2 263 265 18712970

[B4] Joseph BK Oral cancer: prevention and detection Med Princ Pract 2002 11 Suppl 1 32 35 12123114 10.1159/000057776

[B5] Wildt J Bundgaard T Bentzen SM Delay in the diagnosis of oral squamous cell carcinoma Clin Otolaryngol 1995 20 1 21 25 10.1111/j.1365-2273.1995.tb00006.x 7788928

[B6] Burzynski NJ Firriolo FJ Butters JM Sorrell CL Evaluation of oral cancer screening J Cancer Educ 1997 12 2 95 99 9229272 10.1080/08858199709528462

[B7] Bouquot JE Common oral lesions found during a mass screening examination J Am Dent Assoc 1986 112 1 50 57 3455995 10.14219/jada.archive.1986.0007

[B8] Ferullo A Silk H Savageau JA Teaching oral health in U.S. medical schools: results of a national survey Acad Med 2011 86 2 226 230 10.1097/ACM.0b013e3182045a51 21169775

[B9] Rankin KV Burznski NJ Silverman S Jr Scheetz JP Cancer curricula in U.S. dental schools J Cancer Educ 1999 14 1 8 12 10328317 10.1080/08858199909528566

[B10] Sciubba JJ Oral leukoplakia Crit Rev Oral Biol Med 1995 6 2 147 160 10.1177/10454411950060020401 7548621

[B11] Folsom TC White CP Bromer L Canby HF Garrington GE Oral exfoliative study. Review of the literature and report of a three-year study Oral Surg Oral Med Oral Pathol 1972 33 1 61 74 10.1016/0030-4220(72)90209-5 4550162

[B12] Mehrotra R The role of cytology in oral lesions: A review of recent improvements Diagn Cytopathol 2011 39 10.1002/dc.21581 21442772

[B13] Mehrotra R Hullmann M Smeets R Reichert TE Driemel O Oral cytology revisited J Oral Pathol Med 2009 38 2 161 166 19213102 10.1111/j.1600-0714.2008.00709.x

[B14] Sciubba JJ Improving detection of precancerous and cancerous oral lesions. Computer-assisted analysis of the oral brush biopsy. U.S. Collaborative OralCDx Study Group J Am Dent Assoc 1999 130 10 1445 1457 10570588 10.14219/jada.archive.1999.0055

[B15] Scheifele C Schmidt-Westhausen AM Dietrich T Reichart PA The sensitivity and specificity of the OralCDx technique: evaluation of 103 cases Oral Oncol 2004 40 8 824 828 10.1016/j.oraloncology.2004.02.004 15288838

[B16] Patton LL Epstein JB Kerr AR Adjunctive techniques for oral cancer examination and lesion diagnosis: a systematic review of the literature J Am Dent Assoc 2008 139 7 896 905 quiz 993-894 18594075 10.14219/jada.archive.2008.0276

[B17] Lingen MW Kalmar JR Karrison T Speight PM Critical evaluation of diagnostic aids for the detection of oral cancer Oral Oncol 2008 44 1 10 2 10.1016/j.oraloncology.2007.06.011 17825602 PMC2424250

[B18] Potter TJ Summerlin DJ Campbell JH Oral malignancies associated with negative transepithelial brush biopsy J Oral Maxillofac Surg 2003 61 6 674 677 10.1053/joms.2003.50136 12796875

[B19] Poate TW Buchanan JA Hodgson TA An audit of the efficacy of the oral brush biopsy technique in a specialist Oral Medicine unit Oral Oncol 2004 40 8 829 834 10.1016/j.oraloncology.2004.02.005 15288839

[B20] Hohlweg-Majert B Deppe H Metzger MC Sensitivity and specificity of oral brush biopsy Cancer Invest 2009 27 3 293 297 10.1080/07357900802266515 19160103

[B21] Bouquot JE Whitaker SB Oral leukoplakia--rationale for diagnosis and prognosis of its clinical subtypes or "phases" Quintessence Int 1994 25 2 133 140 8183979

[B22] Fischer DJ Epstein JB Morton TH Schwartz SM Interobserver reliability in the histopathologic diagnosis of oral pre-malignant and malignant lesions J Oral Pathol Med 2004 33 2 65 70 10.1111/j.1600-0714.2004.0037n.x 14720191

[B23] Abbey LM Kaugars GE Gunsolley JC Intraexaminer and interexaminer reliability in the diagnosis of oral epithelial dysplasia Oral Surg Oral Med Oral Pathol Oral Radiol Endod 1995 80 2 188 191 10.1016/S1079-2104(05)80201-X 7552884

[B24] Lee JJ Hung HC Cheng SJ Factors associated with underdiagnosis from incisional biopsy of oral leukoplakic lesions Oral Surg Oral Med Oral Pathol Oral Radiol Endod 2007 104 2 217 225 10.1016/j.tripleo.2007.02.012 17560138

[B25] Holmstrup P Vedtofte P Reibel J Stoltze K Oral premalignant lesions: is a biopsy reliable? J Oral Pathol Med 2007 36 5 262 266 10.1111/j.1600-0714.2007.00513.x 17448135

[B26] Koch FP Kunkel M Biesterfeld S Wagner W Diagnostic efficiency of differentiating small cancerous and precancerous lesions using mucosal brush smears of the oral cavity-a prospective and blinded study Clin Oral Investig 2010 in press 10.1007/s00784-010-0434-6 20593209

[B27] Eisen D Frist S The relevance of the high positive predictive value of the oral brush biopsy Oral Oncol 2005 41 7 753 755 author reply 756 10.1016/j.oraloncology.2004.10.004 15936979

[B28] Svirsky JA Burns JC Carpenter WM Comparison of computer-assisted brush biopsy results with follow up scalpel biopsy and histology Gen Dent 2002 50 6 500 503 12572180

[B29] Burkhardt A A response to "sensitivity and specificity of oral brush biopsy" by Hohlweg-Majert et al Cancer Invest 2010 28 5 560 561 10.3109/07357900903095763 20450337

[B30] Kosicki DM Riva C Pajarola GF Burkhardt A Gratz KW [OralCDx brush biopsy--a tool for early diagnosis of oral squamous cell carcinoma] Schweiz Monatsschr Zahnmed 2007 117 3 222 227 17425240

[B31] Bhoopathi V Kabani S Mascarenhas AK Low positive predictive value of the oral brush biopsy in detecting dysplastic oral lesions Cancer 2009 115 5 1036 40 10.1002/cncr.24089 19165806

[B32] Mehrotra R Singh MK Pandya S Singh M The use of an oral brush biopsy without computer-assisted analysis in the evaluation of oral lesions: a study of 94 patients Oral Surg Oral Med Oral Pathol Oral Radiol Endod 2008 106 2 246 253 10.1016/j.tripleo.2008.02.030 18644521

[B33] Delavarian Z Mohtasham N Mosannen-Mozafari P Pakfetrat A Shakeri MT Ghafoorian-Maddah R Evaluation of the diagnostic value of a Modified Liquid-Based Cytology using OralCDx Brush in early detection of oral potentially malignant lesions and oral cancer Med Oral Patol Oral Cir Bucal 2010 15 5 e671 676 20383114 10.4317/medoral.15.e671

[B34] Levine TS Njemenze V Cowpe JG Coleman DV The use of the PAPNET automated cytological screening system for the diagnosis of oral squamous carcinoma Cytopathology 1998 9 6 398 405 10.1046/j.1365-2303.1998.00126.x 9861532

[B35] Drinnan AJ Screening for oral cancer and precancer - A valuable new technique Gen Dent 2000 48 656 660 12004660

[B36] Svirsky JA Burns JC Page DG Abbey LM Computer-assisted analysis of the oral brush biopsy Compendium 2001 22 99 106 11911069

[B37] Lydiatt DD Cancer of the oral cavity and medical malpractice Laryngoscope 2002 112 5 816 819 10.1097/00005537-200205000-00009 12150612

